# Esophagostomy for percutaneous tube feeding in diaphragm paralysis

**DOI:** 10.1002/ccr3.489

**Published:** 2016-01-26

**Authors:** Ezekiel Wong Toh Yoon

**Affiliations:** ^1^ Department of Internal Medicine (Gastroenterology)Hiroshima Kyoritsu Hospital2‐20‐20 Nakasu, Asaminami‐kuHiroshima CityJapan

**Keywords:** Diaphragm paralysis, enteral nutrition, esophagostomy, percutaneous transesophageal gastro‐tubing

## Abstract

PTEG is a minimally invasive method to access the stomach or small intestine via an esophagostomy. It is used as an alternative to nasogastric tubing for gastric decompression or long‐term enteral nutrition when gastrostomy is not suitable, such as in patients with total gastrectomy or advanced gastric cancer.

A 77‐year‐old woman was admitted for severe pneumonia and respiratory failure. She developed dysphagia from disuse syndrome and oral intake was not recommended. However, due to a paralyzed left diaphragm of unknown etiology, percutaneous endoscopic gastrostomy (PEG) for long‐term enteral nutrition was not possible (Fig. 1).

**Figure 1:**
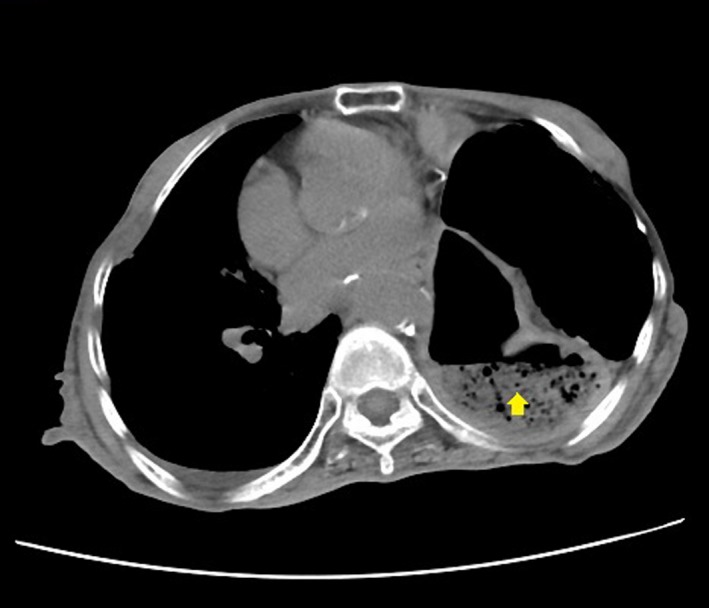
CT scan showing left diaphragm paralysis with the stomach (arrow) within the thoracic cage.

## Is nasogastric tubing the only option?

**Figure 2:**
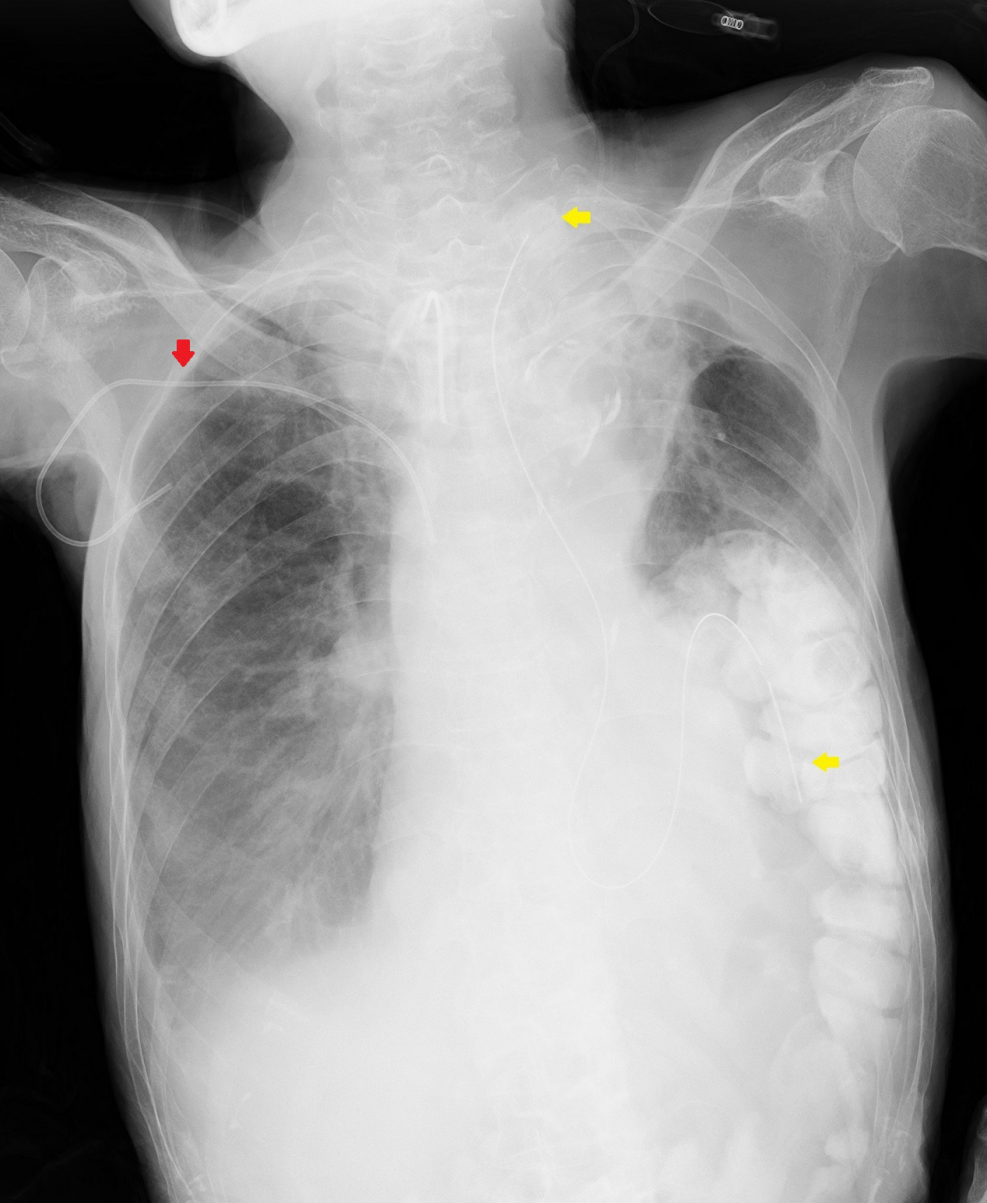
Chest X‐ray after tube placement revealing the PTEG tube (yellow arrows) and a previously inserted central venous line (red arrow).

Instead of nasogastric tubing which is usually meant for the short term, a minimally invasive esophagostomy procedure, PTEG (Percutaneous transesophageal gastro‐tubing), was performed. After inserting a balloon catheter into the upper esophagus (either orally or nasally), a guidewire was introduced into it by puncturing the left side of the neck with an 18G needle under ultrasonography guidance. The puncture site was sufficiently dilated and a 15Fr feeding tube was then inserted into the stomach through the esophagus (Fig. 2). This method, executed with the use of fluoroscopy/endoscopy, was created for bowel decompression and enteral feeding when PEG is not suitable, such as in patients with total gastrectomy, advanced gastric cancer, or massive ascites 1.

## Conflict of Interest

None declared.
